# Detection of *Drechslera avenae* (Eidam) Sharif [*Helminthosporium avenae* (Eidam)] in Black Oat Seeds (*Avena strigosa* Schreb) Using Multispectral Imaging

**DOI:** 10.3390/s20123343

**Published:** 2020-06-12

**Authors:** Fabiano França-Silva, Carlos Henrique Queiroz Rego, Francisco Guilhien Gomes-Junior, Maria Heloisa Duarte de Moraes, André Dantas de Medeiros, Clíssia Barboza da Silva

**Affiliations:** 1 Department of Crop Science, University of São Paulo-Luiz de Queiroz College of Agriculture, 11 Pádua Dias Avenue, 13418-900 Piracicaba, Brazil; carlosqueirozagro@gmail.com (C.H.Q.R.); francisco1@usp.br (F.G.G.-J.); 2 Department of Plant Pathology and Nematology, University of São Paulo-Luiz de Queiroz College of Agriculture, 11 Pádua Dias Avenue, Piracicaba 13418-900, Brazil; mhdmorae@usp.br; 3 Department of Agronomy, Universidade Federal de Viçosa, Peter Henry Rolfs Avenue, Viçosa MG 36570-900, Brazil; andre.d.medeiros@ufv.br; 4 Laboratory of Radiobiology and Environment, University of São Paulo-Center for Nuclear Energy in Agriculture, 303 Centenário Avenue, Piracicaba SP 13416-000, Brazil; clissia_usp@hotmail.com

**Keywords:** machine vision, *Pyrenophora avenae*, reflectance, seed quality, seed pathology

## Abstract

Conventional methods for detecting seed-borne fungi are laborious and time-consuming, requiring specialized analysts for characterization of pathogenic fungi on seed. Multispectral imaging (MSI) combined with machine vision was used as an alternative method to detect *Drechslera avenae* (Eidam) Sharif [*Helminthosporium avenae* (Eidam)] in black oat seeds (*Avena strigosa* Schreb). The seeds were inoculated with *Drechslera avenae* (*D. avenae*) and then incubated for 24, 72 and 120 h. Multispectral images of non-infested and infested seeds were acquired at 19 wavelengths within the spectral range of 365 to 970 nm. A classification model based on linear discriminant analysis (LDA) was created using reflectance, color, and texture features of the seed images. The model developed showed high performance of MSI in detecting *D. avenae* in black oat seeds, particularly using color and texture features from seeds incubated for 120 h, with an accuracy of 0.86 in independent validation. The high precision of the classifier showed that the method using images captured in the Ultraviolet A region (365 nm) could be easily used to classify black oat seeds according to their health status, and results can be achieved more rapidly and effectively compared to conventional methods.

## 1. Introduction

In South America, black oats (*Avena strigosa* Schreb) are cultivated as cover crops or for grain/seed production. Black oat seed oil has the potential to be used in food and cosmetic industries due to its great nutritional value and bioactive compounds [1,2]. In Brazil, seed and grain production was estimated at 275,000 and 900,000 tons, respectively, in the 2019 commercial harvest [3,4]. In order to guarantee high productivity, seeds must meet high quality standards, especially considering health attributes. For instance, seed-borne phytopathogenic fungi can be transferred from farm to farm, reducing the physiological potential of plants with huge economic losses in the production system [5,6].

Helminthosporium leaf spot is a common oat disease caused by *Drechslera avenae* (Eidam) Sharif [*Helminthosporium avenae* (Eidam)], which spreads spores quickly with leaf spot formation and can cause leaf and seedling blight. Serious infections are often responsible for losses in yield and quality of seed and straw and facilitate the occurrence of aflatoxin-producing fungi with further deterioration of seeds [7,8]. *D. avenae* has been reported in all oat-growing areas worldwide, and under condition of high humidity and temperatures from 10 to 22 °C, symptoms of leaf blight may reach up to 100% of plants, with a reduction from 10% to 40% in yield [5,6,9,10].

Currently, the detection of seed-borne fungi is based on visual examination of dry seed, washing test, incubation methods, spore count, or seedling symptoms test [11]. In general, these methods are laborious and time-consuming, requiring specialized analysts. Therefore, rapid, accurate and non-subjective analytical techniques are highly desired for seed health analysis. Advanced spectral sensors combined with computer vision can provide automation and fast assessment of seed health by extraction of spectral, color, and texture features [12].

Multispectral imaging (MSI) is a recent technology that combines traditional optical spectroscopy and computer vision, and can provide spatial and spectral information on different fungi species. Multispectral images are acquired by illuminating samples with light emitting diodes (LEDs) of known spectra at multiple bandwidths from ultraviolet to infrared regions. Thus, the images obtained consist of individual grayscale sub-images, each taken at a predefined spectral band. Reflectance data are registered from a monochromatic image sensor (CCD = charge-coupled device), with simultaneous measurements of multiple components based on chemical composition, color, and texture without sample preparation or destruction. Therefore, MSI systems can represent potential tools for seed health analysis in the agricultural industry, providing important results within a short processing time [11,12,13,14].

MSI has shown promising results in different aspects of seed health, with rapid identification of several fungal species, including *Verticillium* spp., *Fusarium* spp., *Stemphylium botryosum*, *Cladosporium* spp., and *Alternaria alternata* in spinach [15]; *Fusarium* spp. in wheat [13,16]; and *Alternaria infectoria*, *Dothideomycetes* sp., *Fusarium graminearum*, *Fusarium avenaceum*, and *Mycosphaerella tassiana* in barley [12]. Considering that different fungal species have individual spectral characteristics, an MSI system was combined with machine vision to detect *D. avenae* in black oat seeds.

## 2. Materials and Methods

### 2.1. Seed Material and Fungal Inoculation

*D*. *avenae* spores were isolated from black oat seeds of ‘Embrapa 29’ cultivar using the deep-freezing blotter method. The seeds were placed in a 9-cm plastic Petri dish (25 seeds per Petri dish), containing three layers of sterilized blotting paper moistened with sterile distilled water (quantity of 2.5 times the dry-paper weight), kept at 20 °C ± 1 °C for 24 h. Then, seeds were transferred to a freezer at −20 °C for 24 h and, subsequently, incubated at 20 °C ± 2 °C with a photoperiod of 12 h with fluorescent lamps, for 7 days. After this period, the seeds were examined individually with a stereomicroscope, and the identification of *D. avenae* was based on morphological characteristics.

An agar culture medium was used for growing *D. avenae.* The medium was poured into three 15-cm plastic Petri dishes, and after solidification, mycelium fragments were collected from seed surface and transferred to the center of plates using a sterile needle. The plates were kept at 20 °C ± 2 °C in a photoperiod of 12 h with fluorescent lamps, for 10 days.

After complete colonization of each Petri dish by mycelial growth, the plates received a total of 300 seeds. Seeds were previously disinfected for 3 min in 1% sodium hypochlorite solution and washed in distilled water. Afterwards, the seeds were arranged in a single layer on paper towels inside plastic trays, and kept at room temperature for 24 h. After this period, the plates were kept at 20 °C ± 2 °C in a photoperiod of 12 h with fluorescent lamps for 24, 72 and 120 h. Thereafter, seeds were transferred to a plastic tray and arranged in a single layer on paper towels, at room temperature for 24 h [17].

### 2.2. MSI—Image Acquisition and Analysis

Multispectral images of 200 non-infested seeds and 200 infested seeds with *D. avenae* for each period inoculation at 24, 72 and 120 h were captured at 19 wavelengths from ultraviolet (UVA) to NIR (365, 405, 430, 450, 470, 490, 515, 540, 570, 590, 630, 645, 660, 690, 780, 850, 880, 940, 970 nm), using the VideometerLab4^®^ instrument (Videometer A/S, Herlev, Denmark). Four repetitions of 50 seeds per treatment were placed in 9-cm glass Petri dishes containing double-sided adhesive tape for fixing the seeds one by one in a single layer and equidistant. Each plate was positioned at the bottom of the integrating sphere and, after successive illumination of the sample with 19 contiguous LEDs (sequential strobes), a monochromatic image sensor (CCD) registered the reflectance intensities, generating 19 high-resolution images (2192 × 2192 pixels) in one sequence during 5 s.

Data analyses were performed with VideometerLab4 software version 3.14.9 (Videometer A/S, Herlev, Denmark). The multispectral images were transformed using normalized canonical discriminant analysis (nCDA) to minimize the distance within classes and to maximize the distance among classes. Each seed was identified as a region of interest (ROI), and it was built a mask to segment the seeds from the background, which was based on an nCDA transformation of seeds and Petri dish and a simple threshold.

Seeds were collected in a blob database, and 36 variables were extracted from the individual seeds, including tristimulus components of color as hue (angular specification for color perceived as red, yellow, blue or green) and saturation (degree of difference between the color and neutral gray). Color features were extracted from the 19 multispectral images. Multispectral data were transformed to color data by using models for color description, i.e., RGB, XYZ and L*a*b* models: RGB and XYZ models were converted into L*a*b* to obtain more information on color. Texture features were calculated on individual spectral bands and bands derived from these, RGB and CIELab; CIE represents the color space.

MultiColorMean feature extracts the reflectance mean of each seed for the 19 spectral bands from 365 to 970 nm. To eliminate the influence of outliers at both the high and low ends, a trimmed mean excludes 10% of the lowest and highest values before calculating the mean. RegionMSI_Mean calculates a trimmed mean of transformed pixel values within the blob (each single seed), and RegionMSIthresh measures the percentage of blob region with transformation value higher than threshold, based on the nCDA model (derived from all the classes).

A gray level run length matrix (GLRLM) was generated to identify and distinguish texture patterns. GraylevelRunStatistics feature captures the coarseness of a texture in specified directions according to an algorithm described by Galloway [18] and Albregtsen et al. [19]: First = Short Run Emphasis (SRE) measures the distribution of short runs, and higher values indicate fine textures; Second = Long Run Emphasis (LRE) measures the distribution of long runs, and higher values indicate coarse textures; Third = Gray Level Non-Uniformity (GLN) measures the similarity of gray level values in the image, and GLN values are lower if gray level values are similar throughout the image; Fourth = Run Length Non-Uniformity (RLN) expresses the similarity of run lengths throughout the image, with lower values if the run lengths are the same throughout the image; Fifth = Run Percentage (RP) determines the distribution and homogeneity of runs in an image in a particular direction. The texture features described by Chu et al. [20] were also measured: Sixth = Low Grey Level Run Emphasis (LGRE) and Seventh = High Grey Level Run Emphasis (HGRE). Short run emphasis measures the short run distribution and it is large for fine textures. Long run emphasis calculates the long run distribution and it is large for coarse structural textures.

The CIE color spaces were measured for the axes of lightness (L*) and chromaticities (a* and b*), where CIELab L* represents lightness from black to white, CIELab a* the color appearance from green to red and CIELab b* the color appearance from blue to yellow. An intensity-hue-saturation transformation was applied to map the standardized RGB (sRGB) image into intensity, which is independent of color hue that is the dominant wavelength, and saturation which is the colorfulness or the prominence of the dominant color.

### 2.3. Inoculum Verification

In order to confirm that the parameters extracted from the multispectral images were related to presence of *D. avenae*, following the MSI each seed class, 0, 24, 72 and 120 h, was divided into eight repetitions of 25 seeds and incubated at 20 °C ± 2 °C with a photoperiod of 12 h using fluorescent lamps for 5 days. The seeds were examined individually with a stereomicroscope and the identification of *D. avenae* was based on morphological characteristics.

### 2.4. Models for Seed Health Classification

**Figure 1:**
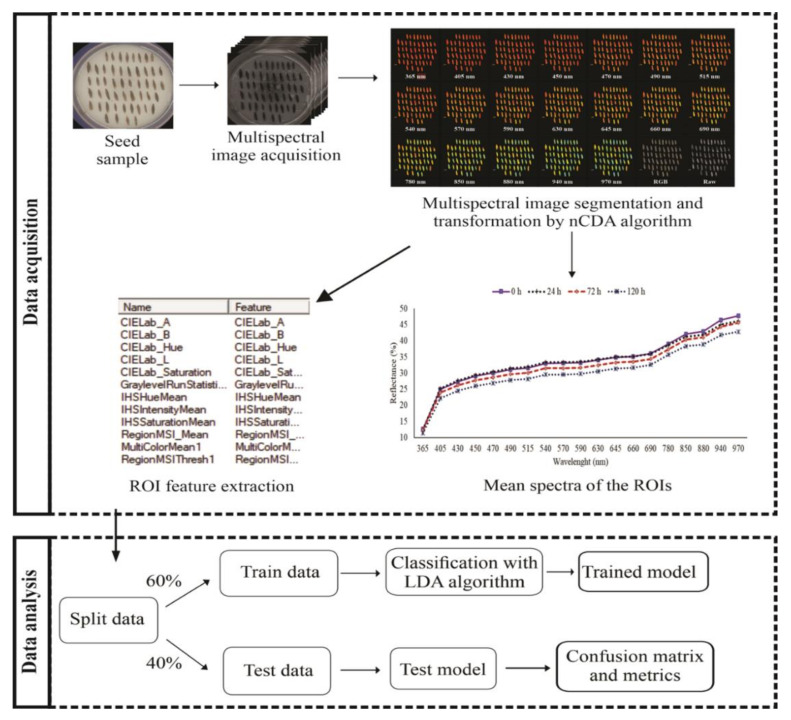
Overall flowchart of the main procedures for multispectral data acquisition and analysis. nCDA-Normalized Canonical Discriminant Analysis. LDA-Linear Discriminant Analysis. ROI-Region Of Interest.

Figure 1 illustrates the critical procedure for analyzing multispectral imaging data. Spectral information obtained from multispectral data were used for developing two models based on Linear Discriminant Analysis (LDA) that can discriminate infested seeds from non-infested seeds. The first model was based on reflectance data obtained for each seed at 19 different wavelengths. The second model was created with color and texture parameters. The data obtained from multispectral images were arranged in an X-matrix (predictor variables) and the values obtained from each seed class at 0, 24, 72 and 120 h, in a Y vector (response variables). In total, 800 seeds, i.e., 200 seeds from each class were used to create a model training/validation. The training set comprised 120 seeds of each class, i.e., 60% of each sample, and the remaining 80 seeds (40% of each sample) comprised the validation set: training set n = 480, testing set n = 320. Cohen’s kappa coefficient and accuracy were used to evaluate the performance of the models. The statistical analyses were performed by VideometerLab4 software, version 3.14.9, and using R software version 3.6.1 [21].

## 3. Results

### 3.1. Spectral Overview of Healthy and Unhealthy Black Oat Seeds

**Figure 2:**
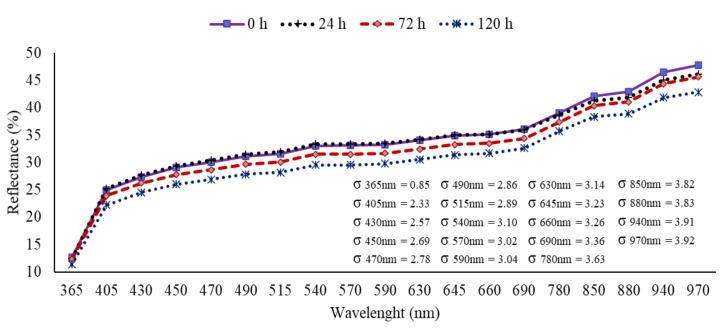
Mean spectral reflectance signatures measured at 19 wavelengths for non-inoculated seeds (0 h) and inoculated seeds with *Drechslera avenae* (Eidam) Sharif, at 24, 72 and 120 h after inoculation. σ represents the standard deviation (+/−) of reflectance data in each wavelength.

The mean reflectance spectra of 200 non-infested seeds and 200 infested seeds with *D. avenae* for each period inoculation at 24, 72 and 120 h obtained at 19 wavelengths are shown in Figure 2. The results of the reflectance spectrum from the NIR region (≥ 850 nm) showed consistent differences between uninoculated and inoculated seeds. At shorter wavelengths, the MSI showed low separation of uninoculated seeds from inoculated seeds for 24 h, but clearly enabled discrimination of seeds inoculated for longer periods at 72 and 120 h. Moreover, there were lower standard deviations at shorter wavelengths, with the lowest value in the UVA region at 365 nm (± 0.85). In general, healthy seeds showed the highest reflectance intensity, with a decrease in the reflectance mean as the inoculation period increased.

**Figure 3:**
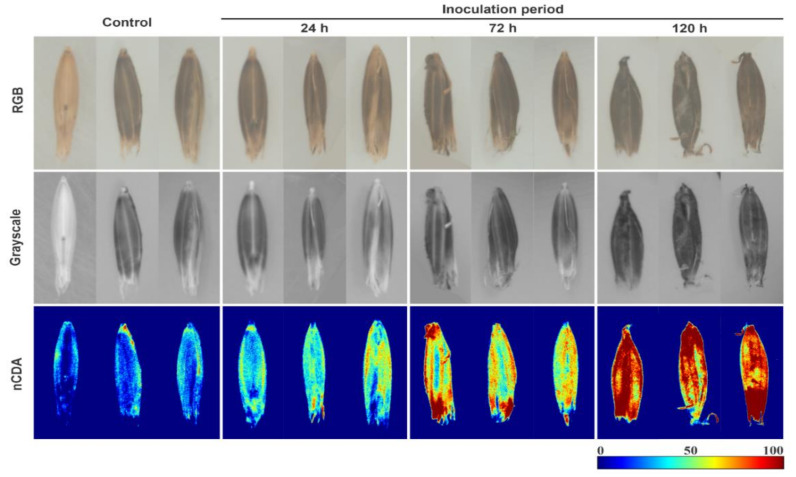
Raw images and corresponding grayscale and nCDA images of black oat seeds at 365 nm for fungus-free seeds (control), and seeds exposed to *Drechslera avenae* (Eidam) Sharif for 24, 72 and 120 h. In the images transformed by nCDA algorithm, blue color represents healthy tissues, green and yellow colors are intermediate contamination, and red color indicates higher fungal contamination.

The reflectance patterns of seed glumellas were evaluated based on pixel intensity of the multispectral images, which were different depending on the inoculation period with *D. avenae* at 0, 24, 72, and 120 h (Figure 3). Overall, after image transformation by nCDA algorithm, the *Helminthosporium* spots were more evident, particularly for extended inoculation periods, meanwhile, it was difficult to detect changes in the seed glumellas from non-transformed RGB and grayscale images, regardless of inoculation period.

### 3.2. Seed Health Classification

Two models based on the LDA algorithm were developed and compared to classify the health status of black oat seeds. The first model used reflectance resources from 19 spectral bands, with an overall accuracy of 0.80 and 0.73 for training and validation sets, respectively (Table 1). Seeds infested for 120 h had 0.84 and 0.86 hit rate for training and testing sets, respectively. On the other hand, there was no distinction between uninoculated and inoculated seeds for 24 h, in which at least 19% of uninoculated seeds were confused with inoculated seeds for 24 h in the testing set, while the confusion increased to 22% in the opposite direction.

In the second model, color and texture descriptors were used to classify seed health status (Table 2). This model presented an overall accuracy of 0.88 and 0.86 for training and validation sets, respectively. The hit rate remained higher in the inoculated seeds for 120 h (0.96 training and 1.00 testing), whereas classes of uninoculated seeds and seeds inoculated for 72 h achieved a hit rate of 0.82 in the testing set. A considerable number of false-positives and false-negatives were shown for seeds inoculated for 24 h, but slightly lower compared to the model based on reflectance descriptors.

**Figure 4:**
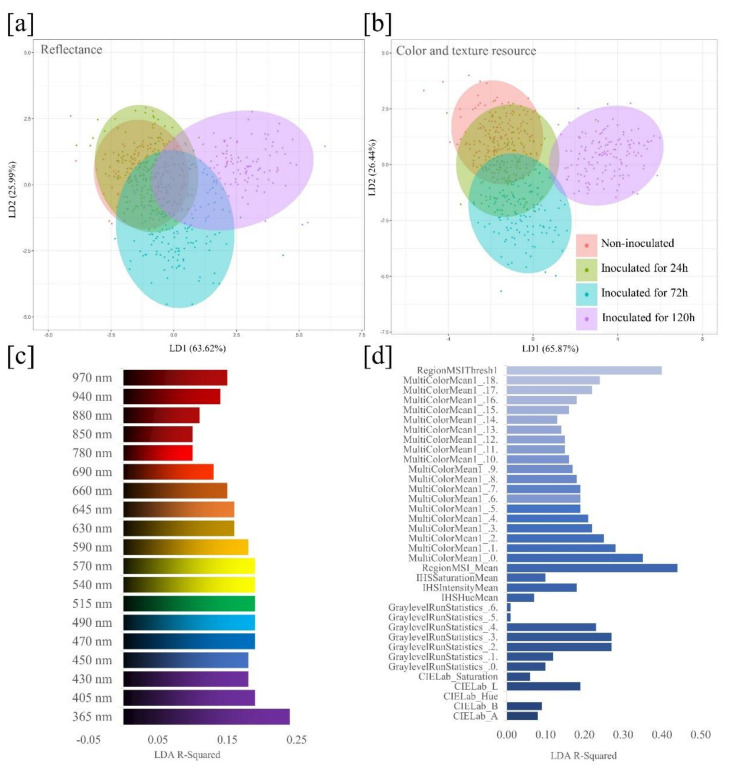
Linear discriminant analysis (LDA) score plot based on reflectance (**a**) and color and texture resources (**b**) of black oat seeds for classes of uninoculated and inoculated seeds with *Drechslera avenae* (Eidam) Sharif. (**a, b**) Ellipses show 95% confidence intervals for each seed health class. For each class, n = 200. (**c**) R-squared values indicate the spectral reflectance contributions of 19 wavelengths, and (**d**) the individual contribution of 36 variables extracted from multispectral images for classification of four seed health classes: 1-uninoculated; 2-inoculated for 24 h; 3-inoculated for 72 h; 4-inoculated for 120 h.

Two discriminant functions, LD1 and LD2, were chosen for analysis, which explained 89.61% and 92.31% of the total variance, respectively, for the model based on reflectance (Figure 4a) and color and texture resources (Figure 4b). Plotting these two discriminant functions resulted in a distinction among the four seed health classes. The model based on color and texture resources were more effective in discriminating health classes, which suggests that these variables are good predictors for classification of black oat seed health status. In general, seeds inoculated for 120 h were better explained by the two models developed.

The contribution histogram shown in Figure 4c indicates the most informative wavelengths based on the coefficient of determination, R-squared, considering the 19 different wavelengths. The most meaningful wavelength for distinguishing uninoculated seeds from seeds inoculated with *D. avenae* was 365 nm, while longer wavelengths showed a trend toward lower R-squared values. Plotting a histogram for the 36 variables extracted from the individual seeds (Figure 4d) allowed to visualize the contribution of each variable to classify the health status of black oat seeds. In fact, the RegionMSI_Mean and RegionMSIthresh were the most meaningful features.

## 4. Discussion

In the present study, we present a new method to health analysis of black oat seeds based on MSI combined with machine vision. Our studies included the application of an nCDA model as a supervised transformation building method, combining spectral data from different seed health classes with their corresponding reflectance, color, and texture parameters. The extraction of multispectral information from each seed enabled the development of accurate models to classify the seeds according to their health status. The nCDA algorithm and statistical modeling were essential to inform the most meaningful wavelength in distinguishing uninoculated seeds from seeds inoculated with *D. avenae* (365 nm). This information would not be solely reached by non-transformed images, especially RGB images (Figure 3), which are limited to the visible light spectrum. Shahin and Symons [22] using MSI associated with chemometric (PLRS) found that wavelengths of 494, 578, 639, and 678 nm show good performance in predicting Canadian wheat seeds contaminated by *Fusarium* spp., with an accuracy of 0.90. The selection of bandwidths of interest is an important phase towards the development of a relatively simple, effective, and accessible system.

The spectral differences among the four seed classes, particularly over longer period of inoculation at 120 h, can be attributed to the mycelial concentration on seeds in addition to biochemical changes caused by defense enzymes that promote seed coat darkening [23]. The increase in color darkening showed in RGB images (Figure 3) may be attributed to oxidative degradation of polyphenol compounds that leads the formation of dark-colored melanin polymers [23]. As a consequence, there were considerable variations in lightness values (CIELab L*), as well as reflectance intensity (MultiColorMean) and texture (GraylevelRunStatistics), facilitating the discrimination between healthy and unhealthy seeds (Figure 4). It is important to emphasize that 100 percent of black oat seeds were contaminated with *D. avenae* after inoculation for 24, 72 and 120 h, and the longer the seed contact with *D. avenae*, the higher the amount of fungal structures, with spores and mycelia covering the seeds.

Reflectance signatures of black oat seeds were different in the absence or presence of *D. avenae*. Healthy seeds showed the highest reflectance intensity, and decreased for extended inoculation periods with *D. avenae*. Light is absorbed to a different extent at parts of increased roughness compared to flat surface parts [24]. Thus, the different reflectances showed for different seed health classes may also be explained by increased surface roughness due to fungal infestation. Furthermore, there are evidences that UV images can provide spatial information on texture variations [25] as well as physical surface properties such as intactness and surface density profiles [24]. In the NIR region, the distinctive spectral patterns correspond to the energy absorption of functional groups containing a hydrogen atom (combination of C-H, N-H and O-H). For instance, the wavelengths 890 and 940 nm are associated with fat, 970 nm with water and the wavelengths related to absorbance of green, yellow and orange color, at 505, 525, 570, and 590 nm, are related to the presence of anthocyanin pigments [26,27].

Previous studies have demonstrated the great potential of MSI for detection of *Fusarium* spp. in wheat [22,28], spinach [15], and maize [29]. The potential of MSI in discriminating seed-borne fungi, although influenced by other factors, is closely related to physical and chemical changes induced by pathogens [30,31,32].

## 5. Conclusions

Our findings indicate that MSI technique was effective for identifying *D. avenae* in black oat seeds. The color and texture features produced better predictive accuracy of 0.86 and correct prediction of 0.78, 0.83 and 1.00 for classes of seeds inoculated for 24, 72 and 120 h, respectively. MSI can be a useful tool to assure black oat seeds are not carrying important pathogens.

## Figures and Tables

**Table 1:** Confusion matrices of the LDA model in training and testing sets using reflectance data of black oat seeds obtained at 19 wavelengths (365 to 970 nm) for class membership of non-inoculated (NI) and inoculated seeds (I) with *Drechslera avenae* (Eidam) Sharif for 24, 72 and 120 h.

Treatment^1^	Training Set (n = 480)				
	NI^a^	I.^b^ 24 h	I. 72 h	I. 120 h	% correct
NI	98	18	3	1	81.67
I. 24 h	16	93	11	0	77.50
I. 72 h	7	11	93	9	77.50
I. 120 h	5	3	11	101	84.17
Overall Accuracy	0.80				
Kappa	0.74				
**Treatment**	**Testing Set (n = 320)**				
	**NI^a^**	**I.^b^ 24 h**	**I. 72 h**	**I. 120 h**	**% correct**
NI	58	19	2	1	72.50
I. 24 h	22	41	15	2	51.25
I. 72 h	3	6	66	5	82.50
I. 120 h	1	2	8	69	86.25
Overall Accuracy	0.73				
Kappa	0.64				

^1^ In the rows are the true seed classes, and in the columns are the estimated classes. ^a^ NI: non-inoculatedseeds; ^b^ I: seeds inoculated with *Drechslera avenae* (Eidam) Sharif.

**Table 2:** Confusion matrices of the LDA model in training and testing sets using color and texture resources of black oat seeds obtained at 19 wavelengths (365 to 970 nm) for class membership of non-inoculated (NI) and inoculated seeds (I) with *Drechslera avenae* (Eidam) Sharif for 24, 72 and 120 h.

Treatment ^1^	Training Set (n = 480)				
	NI ^a^	I. ^b^ 24 h	I. 72 h	I. 120 h	% correct
NI	103	16	1	0	85.83
I. 24 h	12	101	6	1	84.17
I. 72 h	3	9	104	4	86.67
I. 120 h	0	1	3	116	96.67
Overall Accuracy	0.88				
Kappa	0.84				
**Treatment**	**Testing Set (n = 320)**				
	**NI ^a^**	**I. ^b^ 24 h**	**I. 72 h**	**I. 120 h**	**% correct**
NI	66	12	2	0	82.5
I. 24 h	8	62	10	0	77.5
I. 72 h	1	12	66	1	82.5
I. 120 h	0	0	0	80	100
Overall Accuracy	0.86				
Kappa	0.81				

^1^ In the rows are the true seed classes, and in the columns are the estimated classes. ^a^ NI: non-inoculated seeds; ^b^ I: seeds inoculated with *Drechslera avenae* (Eidam) Sharif.

## References

[1] Martínez-Villaluenga C., Peñas E. (2017). Health benefits of oat: Current evidence and molecular mechanisms. Curr. Opin. Food Sci..

[2] Heuzé V., Tran G., Hassoun P., Lebas F. (2015). Black oat (Avena strigosa). Feedipedia, a programme by INRA, CIRAD, AFZ and FAO. https://www.feedipedia.org/node/581.

[3] Kist B.B., Santos C.E., Carvalho C., Beling R.R. (2019). Anuário brasileiro de sementes 2019. http://www.editoragazeta.com.br/sitewp/wp-content/uploads/2019/08/SEMENTES_2019.pdf.

[4] Companhia Nacional de Abastecimento (2018). Acompanhamento da safra brasileira de grãos: Safra 2019–2020. https://www.conab.gov.br/component/k2/item/download/31802_7ba8b57a67345b0bf2f9c691cd65fdf6.

[5] Carmona M.A., Zweegman J., Reis E.M. (2004). Detection and transmission of *Drechslera avenae* from oat seed. Fitopatol. Bras..

[6] Lângaro N.C., Reis E.M., Floss E.L. (2001). Detection of *Drechslera avenae* in oat seeds. Fitopatol. Bras..

[7] Tola M., Kebede B. (2016). Occurrence, importance and control of mycotoxins: A review. Cogent Food Agric..

[8] Husseina L.T., Saadullaha A.A.M. (2018). Mycoflora and incidence of aflatoxin in wheat seeds from Duhok province, Kurdistan region of Iraq. Sci. J. Univ. Zakho..

[9] Atri A., Tiwana U.S. (2019). Effect of seed treatment and foliar spray on leaf blight of fodder oat in Punjab. Phytoparasitica.

[10] Pille S., Mati K. (2011). Timing of fungicide application for profitable disease management in oat (*Avena sativa* L.). Zemdirbyste.

[11] ElMasry G., Mandour N., Al-Rejaie S., Belin E., Rousseau D. (2019). Recent applications of multispectral imaging in seed phenotyping and quality monitoring—An Overview. Sensors.

[12] Boelt B., Shrestha S., Salimi Z., Jørgensen J.R., Nicolaisen M., Carstensen J.M. (2018). Multispectral imaging—A new tool in seed quality assessment?. Seed Sci. Res..

[13] Jørgensen J.R., Shrestha S. Detection of Fusarium in wheat by multispectral Imaging. Proceedings of the Final COBRA Conference.

[14] Shrestha S., Deleuran L.C., Olesen M.H., Gislum R. (2015). Use of Multispectral imaging in varietal identification of tomato. Sensors.

[15] Olesen M.H., Carstensen J.M., Boelt B. (2011). Multispectral imaging as a potential tool for seed health testing of spinach (*Spinacia oleracea* L.). Seed Sci. Technol..

[16] Vrešak M., Olesen M.H., Gislum R., Bavec F., Jørgensen J.R. (2016). The use of image-spectroscopy technology as a diagnostic method for seed health testing and variety identification. PLoS ONE.

[17] Silva F.F., Castro E.M., Moreira S.I., Ferreira T.C., Lima A.E., Alves E. (2017). Emergence and ultrastructural analysis of soybean seedlings inoculated with *Sclerotinia sclerotiorum* under the application of *Trichoderma harzianum*. Summa Phytopathol..

[18] Galloway M.M. (1975). Texture analysis using gray level run lengths. Comput. Graph. Image Process..

[19] Albregtsen F., Nielsen B. (2000). Texture classification based on cooccurrence of gray level run length matrices. Aust. J. Intell. Info. Process. Syst..

[20] Chu A., Sehgal C.M., Greenleaf J.F. (1990). Use of gray value distribution of run lengths for texture analysis. Pattern Recognit. Lett..

[21] R Core Team (2019). R: A language and environment for statistical computing. http://www.r-project.org/index.html.

[22] Shahin M.A., Symons S.J. (2012). Detection of Fusarium damage in Canadian wheat using visible/near-infrared hyperspectral imaging. J. Food Meas. Charact..

[23] Fuerst E.P., Okubara P.A., Anderson J.V., Morris C.F. (2014). Polyphenol oxidase as a biochemical seed defense mechanism. Front. Plant Sci..

[24] Klukkert M., Wu J.X., Rantanen J., Carstensen J.M., Rades T., Leopold C.S. (2016). Multispectral UV imaging for fast and non-destructive quality control of chemical and physical tablet attributes. Eur. J. Pharm. Sci..

[25] Jaillais B., Perrin E., Mangavel C., Bertrand D. (2011). Characterization of the desiccation of wheat kernels by multivariate imaging. Planta.

[26] Jue T., Masuda K. (2013). Application of Near Infrared Spectroscopy in Biomedicine.

[27] Sendin K., Manley M., Williams P.J. (2018). Classification of white maize defects with multispectral imaging. Food Chem..

[28] Bauriegel E., Giebel A., Geyer M., Schmidt U., Herppich W.B. (2011). Early detection of Fusarium infection in wheat using hyper-spectral imaging. Comput. Electron. Agric..

[29] Del Fiore A., Reverberi M., Ricelli A., Pinzari F., Serranti S., Fabbri A.A., Bonifazie G., Fanelli C. (2010). Early detection of toxigenic fungi on maize by hyperspectral imaging analysis. J. Food Microbiol..

[30] Dammer K.H., Möller B., Rodemann B., Heppner D. (2011). Detection of head blight (*Fusarium* sp.) in winter wheat by color and multispectral image analyzes. Crop Prot..

[31] Jaillais B., Roumet P., Pinson-Gadais L., Bertrand D. (2015). Detection of Fusarium head blight contamination in wheat kernels by multivariate imaging. Food Control..

[32] Shrestha S., Knapič M., Žibrat U., Deleuran L.C., Gislum R. (2016). Single seed near-infrared hyperspectral imaging in determining tomato (*Solanum lycopersicum* L.) seed quality in association with multivariate data analysis. Sens. Actuators B Chem..

